# Compliance with NICE guidelines when commissioning varicose vein procedures

**DOI:** 10.1002/bjs5.95

**Published:** 2018-08-29

**Authors:** D. Carradice, J. Forsyth, A. Mohammed, C. Leung, L. Hitchman, A. E. Harwood, T. Wallace, G. E. Smith, B. Campbell, I. Chetter

**Affiliations:** ^1^ Academic Vascular Surgical Unit Hull York Medical School and Hull Royal Infirmary Hull UK; ^2^ Department of Vascular Surgery Royal Devon and Exeter Hospital (Wonford) Exeter UK

## Abstract

**Background:**

Varicose veins impair quality of life and can lead to chronic leg ulcers. National Institute for Health and Care Excellence (NICE) guidelines (CG168) set out evidence‐based standards for patient management. In England, Clinical Commissioning Groups (CCGs) fund NHS care within their locality. The objective of this study was to evaluate CCGs' commissioning policies and compare them with CG168.

**Methods:**

Searches were made for the published policies of all 206 English CCGs. They were reviewed for compliance with NICE guidelines and the associated quality standard. Areas of disagreement were analysed for themes.

**Results:**

Some 203 CCGs (98·5 per cent) had a published policy and 190 (93·6 per cent) of these were published after publication of CG168. Only 73 of the policies (36·0 per cent) were compliant with CG168. Treatment was restricted on the basis of clinical disease severity in 119 CCGs (58·6 per cent); 29 (14·3 per cent) stipulated delay of treatment using a ‘trial’ of conservative treatment; 22 (10·8 per cent) used lifestyle‐related factors such as BMI and smoking status to ration treatment. Treatment was commissioned for uncomplicated symptomatic varicose veins in 87 CCGs (42·9 per cent), but some applied additional rationing mechanisms; 109 CCGs (53·7 per cent) would treat oedema, 183 (90·1 per cent) would treat skin and soft tissue damage, 202 (99·5 per cent) healed ulceration, and all would allow active ulcers to be treated.

**Discussion:**

The majority of CCGs in England have commissioning policies that contradict NICE guidelines. Rationing strategies include disease severity, delay and patient lifestyle‐related factors, creating unwarranted geographical variation for varicose vein treatment, disregarding the NHS Constitution for England, and perhaps leading to an increase in costly treatment of chronic complications in the long term.

## Introduction

Varicose veins (VVs) are the most common manifestation of a spectrum of disease caused by superficial venous incompetence (SVI) of the leg. They stem from inflammatory changes in the vein wall and valve structure that remain poorly understood. The resulting venous incompetence leads to venous hypertension, which produces further inflammatory and structural changes within the veins, causing them to develop their classic bulging, tortuous appearance and progressive incompetence. The venous hypertension can also cause oedema and inflammatory changes in the soft tissues of the lower leg, resulting in permanent skin damage, lipodermatosclerosis and ulceration. The factors governing progression through this spectrum are unclear, and the prognosis for the individual patient is unpredictable.

VVs are extremely common, affecting 40–50 per cent of the adult population^1^, ^2^, ^3^, and many people have no symptoms. This has resulted in a common misconception that they are purely cosmetic, with serious implications for the provision of treatment. When symptoms do occur, they often cause significant limitation in health‐related quality of life (QoL), predominantly in physical domains^4^, ^5^, ^6^, ^7^, ^8^, ^9^, ^10^, ^11^, ^12^, ^13^, ^14^, ^15^, ^16^. For patients who progress to skin damage and ulceration, the consequences are more serious, with major QoL impairment and the need for prolonged and costly nursing care.

Treatment of VVs aims to reduce venous pressure back to normal physiological levels. Typically the incompetent veins are either removed (as in traditional surgical ligation and stripping) or ablated (by endovenous techniques such as endothermal ablation or foam sclerotherapy). Randomized trials comparing the outcomes of the different treatments have shown that they all result in a significant improvement in QoL^17^, ^18^, ^19^, ^20^, ^21^. In addition, there is good evidence that treatment is highly cost‐effective when compared with conservative measures^19^ ^22^.

Despite the evidence of benefit, financial pressures on health services, especially during the last 10 years, have resulted in widespread restrictions in access to treatment in England. Commissioners in different parts of the country have introduced a variety of restrictions to the referral and treatment of patients with VVs. In response to this geographical variation, the National Institute for Health and Care Excellence (NICE) published a Clinical Guideline (CG168) in July 2013^23^ and a Quality Standard (QS67) in August 2014^24^. These recommended that interventional treatment should be offered to patients with symptoms irrespective of the presence of complications such as bleeding and skin changes. These guidelines were formulated by an independent guideline group, using NICE's principles for consideration of all the available evidence, on both clinical outcomes and cost‐effectiveness.

The aim of this study was to establish whether the current commissioning policies in England comply with the evidence‐based NICE guidelines.

## Methods

NHS England has a system of local commissioning for the treatment of VVs. Commissioning decisions are made by local Clinical Commissioning Groups (CCGs) regarding the availability and funding of care in their local areas. Each commissioning area was researched separately by two authors. Their websites were searched between 17 and 24 April 2017 for any published policy on referral and treatment of VVs. Both authors independently noted commissioning criteria from the policy. These were compared and, if there was any disagreement about the precise criteria, a final decision was made by another author. The data were recorded and stored in a Microsoft Excel^®^ spreadsheet (Microsoft, Redmond, Washington, USA), which was also used for data analysis. CCGs with no policy available online were contacted by telephone to ascertain whether they had a policy, and to acquire the policy if there was one. If telephone contact failed, a written freedom of information request was submitted. By law, this requires a response within 20 working days.

The primary outcome measure was whether the policy was fully compliant with NICE CG168 and allowed the routine commissioning of the treatment of suitable patients as per the stated criteria (*Table* 1) without further qualification or limitations. A secondary analysis studied the ways in which the non‐compliant policies differed from NICE CG168.

**Table 1:** Key recommendations from NICE Clinical Guidance CG168 pertaining to commissioners

Referral to a vascular service of patients with
Symptomatic varicose veins
Lower‐limb skin changes thought to be caused by chronic venous insufficiency
Superficial vein thrombosis and suspected venous incompetence
Venous leg ulcer
Healed venous leg ulcer
Treatment for patients with SVI
Offer interventional treatment to ablate or remove diseased veins
Do not offer compression treatment unless unsuitable for interventional treatment

SVI, superficial venous incompetence.

Ethical approval was not required for this study. A venous Public and Patient Involvement (PPI) group was set up in 2015 using a National Institute for Health Research PPI grant; the group supported the importance of this study and reviewed the final draft of the manuscript.

## Results

Some 203 of the 206 CCGs in England (98·5 per cent) had a written policy for the commissioning of treatment for VVs. In 198 (97·5 per cent) the policy was available online. The three CCGs with no policy were in the process of drafting one. Some 190 policies (93·6 per cent) were published after the publication of NICE CG168 in July 2013 and therefore should have been written with knowledge of it.

**Figure 1:**
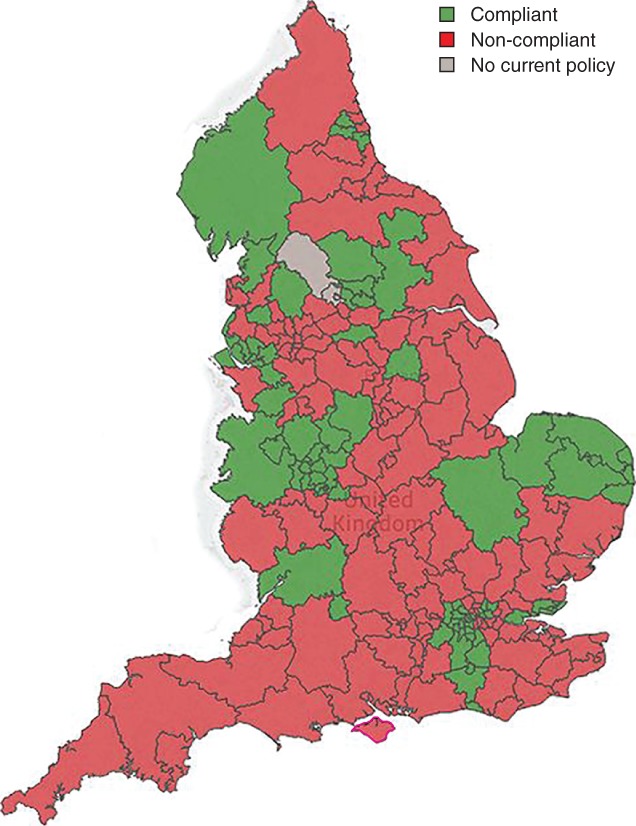
Compliance of Clinical Commissioning Groups in England with NICE guidelines for management of patients with varicose veins

**Figure 2:**
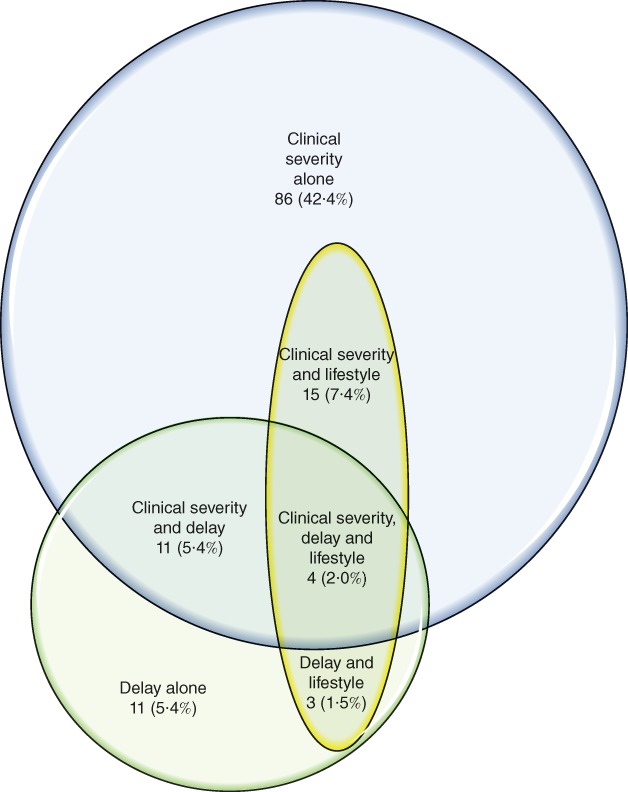
Methods of rationing employed in commissioning policies of 203 Clinical Commissioning Groups in England: clinical severity (blue), delay in treatment (green), lifestyle factors (yellow)

Only 73 (36·0 per cent) of the policies were fully compliant with NICE CG168 (*Fig*. 1). A further 119 CCGs (58·6 per cent) did not comply with the NICE guideline because they limited access to treatment based on clinical disease severity, opting to wait until soft tissue damage or ulceration had occurred. Twenty‐nine policies (14·3 per cent) stipulated a delay in either referral or treatment, or both, for a particular period – often called a ‘trial of conservative treatment’. This typically consisted of compression treatment for 6 months, but the range was 3–12 months. Finally, 22 CCGs (10·8 per cent) chose to ration treatment for patients with certain lifestyle‐related criteria, such as high BMI or smoking. Some policies did not comply with NICE guidelines in more than one respect, as shown in *Fig*. 2.

**Figure 3:**
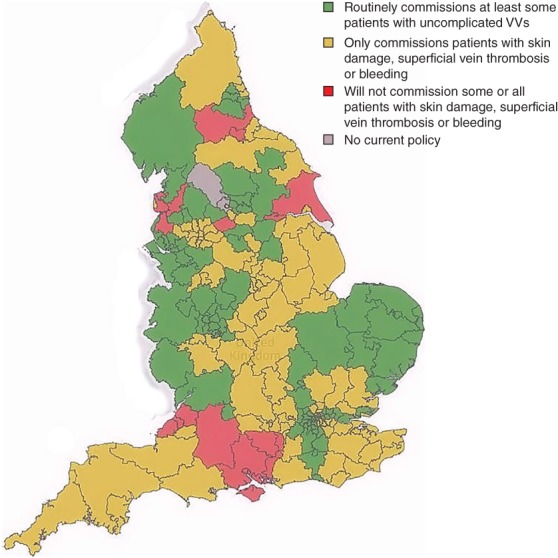
Commissioning policies of Clinical Commissioning Groups in England with regard to clinical severity alone. VV, varicose vein

The most common reason for which policies were non‐compliant was the limitation of treatment to those with greater severity of clinical disease. On this basis, patients with symptomatic uncomplicated VVs had access to treatment within the jurisdiction of only 87 CCGs (42·9 per cent), and patients with oedema in association with their VVs had access in only 109 CCG areas (53·7 per cent). Patients with skin and soft tissue damage had access in 183 CCG areas (90·1 per cent), those with healed ulceration in 202 (99·5 per cent), and those with active ulceration in 203 (100 per cent). These figures apply to clinical severity in isolation; a proportion of policies applied more than one rationing mechanism. For example, 14 of the 87 CCGs treating uncomplicated symptomatic VVs applied additional rationing, by either delay or lifestyle factors, or both (*Fig*. 3).

Some of the policies were explicit about their reasons for non‐compliance with NICE CG168. A common reason given was that implementation of the NICE guideline was ‘too costly’ (11 CCGs). Statements were made that VVs were ‘low priority’ (9 CCGs), and 11 CCGs labelled VV treatment as a ‘cosmetic procedure’. Correspondence with some CCGs indicated that they were considering drafting a more restrictive policy in the near future. From 1 December 2017, a group of CCGs in the North of England changed to a combined policy in which commissioning is limited to the treatment of ulcer disease, but only after a delay of 6 months, and one of these CCGs will not commission treatment for smokers or those with a BMI over 35 kg/m^2^.

## Discussion

NICE guidance is regarded as describing the standard of care for health services in the UK and is it normally expected that its recommendations will be followed by commissioners and providers of healthcare services. NICE guidelines are produced by independent committees including all key stakeholders, using well defined and established processes^25^. During guidance development, each treatment option (including conservative treatment) is considered in terms of safety, efficacy, effectiveness, cost‐effectiveness and acceptability. NICE Clinical Guidelines are aimed primarily at the National Health Service (NHS) in England and Wales, but they have also become respected and influential worldwide. The Clinical Guideline (CG168)^23^ and Quality Standard (QS67)^24^ on VVs make clear recommendations about which patients should be referred to a vascular service and how they should be managed. They were accompanied by implementation tools and resources aimed at supporting healthcare commissioners and hospitals to adopt the recommendations^26^ ^27^.

There is another important consideration regarding the provision of care, and that is the NHS Constitution^28^, which represents the covenant between the government and the people with respect to the conduct of the NHS. It states that the NHS should provide a comprehensive service and that access to services should be based on clinical need and no other factor. Stated rights of a patient are that they will not be refused access on unreasonable grounds, and that they have the right to receive the treatment that is appropriate to them, specifically highlighting treatments that have been recommended by NICE. The results of this study are a direct contradiction of each of these points and represent the emerging differences between the NHS that is promised and the NHS that is delivered.

This study of local commissioning policies across England has identified widespread, conscious non‐compliance with the NICE guidance on which patients may be referred and treated. It has identified three main strategies for limiting access to treatment. The first and most common strategy is to withhold intervention until a later disease stage, for example when skin changes have developed or even when ulceration or bleeding has occurred. This approach is applied variably across the country, as shown in *Figs* 2 and 3, amounting to unwarranted geographical variation of access to treatment.

This strategy ignores the evidence showing that QoL improvement is actually higher when the disease is treated before skin damage is done and recurrence rates also appear to be lower^29^. Disease progression to ulceration has a large impact on QoL, similar to that experienced by patients with end‐stage chronic disease such as congestive cardiac failure or chronic obstructive pulmonary disease^4^ ^30^, ^31^, ^32^, ^33^. Patients' lives must revolve around regular dressing changes. Withholding treatment for the majority of patients with VVs is an untried experiment, and it is unknown exactly how many will progress to ulceration. Venous leg ulcers are the most common type of chronic wound; their management was estimated to cost the NHS around £2 billion per year in 2013–2014^34^. Even a modest increase in prevalence could have significant cost implications that could far outweigh any initial savings.

The second strategy for rationing is by delay, through a period of conservative management before referral or definitive treatment. Evidence suggests that conservative treatment is inferior^19^, hence the specific recommendation by NICE that it be avoided. Accepting this fact, it can be assumed that all patients will need to go on to interventional treatment, and therefore this should not reduce the number of procedures overall. The reality is that this mechanism relies on establishing sufficient barriers between patient and treatment so that some will opt for treatment in the private sector and others will decide to live with their symptoms and tolerate a chronic impairment in QoL and the risk of disease progression.

The third strategy is the introduction of lifestyle‐related criteria, restricting referral because of obesity or smoking. Similar rationing strategies have attracted controversy in the commissioning of orthopaedic lower‐limb procedures^35^, ^36^, ^37^, ^38^. Part of the justification was the risks that obesity and smoking posed for anaesthetic and significant surgical complications, but these considerations are not now relevant for most varicose vein treatments, which are performed using endovenous techniques under local anaesthesia.

In considering the reasons for the widespread non‐compliance with NICE guidance, cost may be the key factor. Some CCGs were explicit that the reason for their non‐compliance was the cost of implementation. The interventional treatment of VVs has been shown to be highly cost‐effective compared with conventional willingness‐to‐pay thresholds^17^ ^22^, and estimated incremental cost‐effectiveness ratios (cost per quality‐adjusted life‐year, QALY) are lower than those of many other treatments routinely offered within the NHS^19^. Health economic theory is that such a treatment should be commissioned and the healthcare programme that offers the highest cost per QALY discontinued. This argument may be countered by affordability. VVs are common; therefore, despite the relatively low cost per patient and per QALY gained, the overall budgetary impact of treating this common condition is sizeable. There has been a move towards increasing consideration of the affordability of healthcare programmes, rather than focusing on the cost per QALY and per patient. This raises an ethical dilemma. A patient's ‘need’ for treatment is proportional to their capacity to benefit from it^39^. Should a patient in need of treatment be denied that treatment purely because of the prevalence of the disease, rather than their capacity to benefit from what is a clinically effective and cost‐effective treatment? This collectivization of societal benefit flies in the face of the NHS Constitution^28^.

The perception of VVs and their implications is an important issue. VVs are widely perceived as primarily a cosmetic problem and they are bundled within cosmetic surgery in commissioning policies. Cosmetic concern is an issue for many people with VVs; however, when they start to give rise to symptoms, these can cause significant impairment in QoL even when relatively modest^4^, ^5^, ^6^, ^7^, ^8^, ^9^, ^10^, ^11^, ^12^, ^13^. This impairment is typically within physical and not psychological domains, which is not consistent with cosmetic conditions, but rather with a physical disease^4^, ^5^, ^6^, ^7^, ^8^, ^9^, ^10^, ^11^, ^12^, ^13^. Symptoms can impact upon activities of daily living, employment and caring roles. There is good evidence from a number of randomized trials^17^, ^18^, ^19^, ^20^, ^21^, which show improvement in these domains following treatment of VVs. Education is therefore needed for commissioners regarding the impact of symptoms on people's lives. Even more importantly, commissioners need to be mindful of the serious consequences of progressive skin changes and ulcers that can be caused by VVs. In addition to this, VVs are thought to be a key aetiological factor in spontaneous superficial vein thrombosis of the leg. This condition has been shown to be associated with a deep vein thrombosis in around 25 per cent of cases^40^, and thromboembolic disease of the deep veins carries well recognized long‐term morbidity and risk of mortality.

The main limitation of this study is that it examined only the published or stated policies of CCGs. In practice, commissioning practices may not concur with published policies; some are more restrictive. One CCG has a policy stating that it will routinely fund the treatment of patients with any skin changes. However, in practice this is not the case and the CCG demands an individual funding request for each patient with a justification required as to their ‘exceptional’ need for treatment. The methodology of this study did not capture this practice.

The NHS is currently under great financial strain and it is not surprising that decisions are being made to restrict services. However, these decisions should be based on a good understanding of the issues and on sufficient public debate. It is clear from this study that at an overall national level there has been no improvement in access to care for people with VVs since the publication of NICE CG168 (and QS67), compared with restrictions documented before that time^41^ ^42^. A pivotal aim when NICE was created in 1999 was to address geographical variations and resulting inequalities in healthcare. The failure of many commissioning policies to observe NICE guidance on referral and treatment is a serious matter. At worst, it means that some patients will develop chronic venous ulcers, which cause great suffering, with a major cost impact on the health service. More generally, it fails to allow access for people with troublesome symptoms to cost‐effective treatments that could improve their QoL. In addressing this situation, better publicity and education are needed for commissioners, healthcare professionals and the public regarding VVs and their consequences.
